# Molecular Epidemiology of Antimicrobial Resistance, Virulence and Capsular Serotypes of Carbapenemase-Carrying *Klebsiella pneumoniae* in China

**DOI:** 10.3390/antibiotics11081100

**Published:** 2022-08-13

**Authors:** Lina Zhao, Xinxin Xia, Ting Yuan, Junying Zhu, Zhen Shen, Min Li

**Affiliations:** 1 Department of Laboratory Medicine, Ren Ji Hospital, Shanghai Jiao Tong University School of Medicine, Shanghai 200127, China; 2 Li Ka Shing Faculty of Medicine, The University of Hong Kong, Hong Kong SAR 999077, China; 3 Faculty of Medical Laboratory Science, College of Health Science and Technology, Shanghai Jiao Tong University School of Medicine, Shanghai 200025, China

**Keywords:** carbapenemase, *Klebsiella pneumoniae*, antimicrobial resistance, virulence, serotype

## Abstract

This study analyzed genomic data of 4643 strains of carbapenemase-carrying *Klebsiella pneumoniae* (KPN) in China by using the Kleborate software package. The data showed rich diversity in carbapenemase-carrying KPN genomes, which contain not only 152 sequence types but also 90 capsular serotypes. In 2013, the transfer of carbapenemase to hypervirulent *Klebsiella pneumoniae* (HvKP) of KL1 and KL2 occurred, and since 2014, the propagation of carbapenemase into mammals, poultry, and insects has been detected. The ST11 capsular serotype had a reversal of the prevalence of KL47 and KL64 in 2016, with KL64 replacing KL47 as the dominant serotype. Colibactin is a very suitable indicator to differentiate KL1-type HvKP and classic Klebsiella pneumoniae. The most prevalent yersiniabactin of KL1 is *ybt1* ICEKp10, and that of ST11 carbapenem-resistant KPN(ST11-CRKP) is *ybt9* ICEKp3. The virulence genes of KL1 carbapenem-resistant hypervirulent KPN (KL1-CRHvKP), as well as ST65- and ST86-type KL2-CRHvKP, were not lost after carbapenemase was obtained.

## 1. Introduction

The *Klebsiella pneumoniae* (KPN) genome has a size of about 5.5 Mbp, encoding 5000~6000 genes, with rich genetic diversity [1]. Classic KPN (CKP) is an opportunistic pathogen that can colonize the human gastrointestinal tract, skin, oral cavity, and nasopharynx [2,3]. CKP usually infects neonates, the elderly, and immunocompromised patients and is the leading cause of hospital-acquired infection and neonatal sepsis [4,5]. In contrast, hypervirulent KPN (HvKP) often infects healthy individuals with normal immunity, causing community-acquired infection. Furthermore, the infections are invasive, including purulent liver abscesses, endophthalmitis, and meningitis [6].

CKP is a key host and transmission vector of clinically important antimicrobial resistance genes [7]. The spread of carbapenem-resistant *Klebsiella pneumoniae* (CRKP) has become an urgent global public health problem [8,9]. HvKP infection is dominated by two lineages: CG23 and CG65 [10,11,12], with CG23 being the most common. Compared with the rich genetic diversity of CKP, the CG23 core chromosome has limited nucleotide variation, and the acquired virulence genes are highly conserved [13,14]. The enhanced or high virulence is associated with specific capsular serotypes, such as KL1 and KL2, and with accessory genes carried by mobile elements [15], such as genotoxin colibactin, as well as yersiniabactin, aerobactin, and salmochelin that encode the additional siderophore system [16,17]. High virulence is also associated with *rmpADC*, which is a hypermucus phenotype-related gene [18,19]. KL1-type HvKP acquiring carbapenemase and ST11-CRKP acquiring HvKP virulence genes appeared in Zhejiang Province, China, in 2015 and 2017, respectively [20,21]. The emergence of a fusion strain CRHvKP, with high virulence and carbapenem resistance, has become a significant clinical problem.

The long-term and nationwide molecular epidemiological monitoring data for carbapenemase-carrying KPN in China are limited. However, with the reduction in whole-genome sequencing costs, the National Center for Biotechnology Information (NCBI) has shared public whole-genome data of bacteria from various countries and regions. Furthermore, analyses of antimicrobial resistance, virulence, and mobile elements, as well as the development and promotion of multiple types of software, have facilitated the analysis of these large data sets. In this study, we downloaded NCBI genome data of 37,322 strains of KPN and conducted a molecular epidemiological analysis of antimicrobial resistance, virulence, and capsular serotypes of carbapenemase-carrying KPN in China. This genomic data set represents the largest and most geographically diverse KPN sets in China to date.

## 2. Results

**Figure 1:**
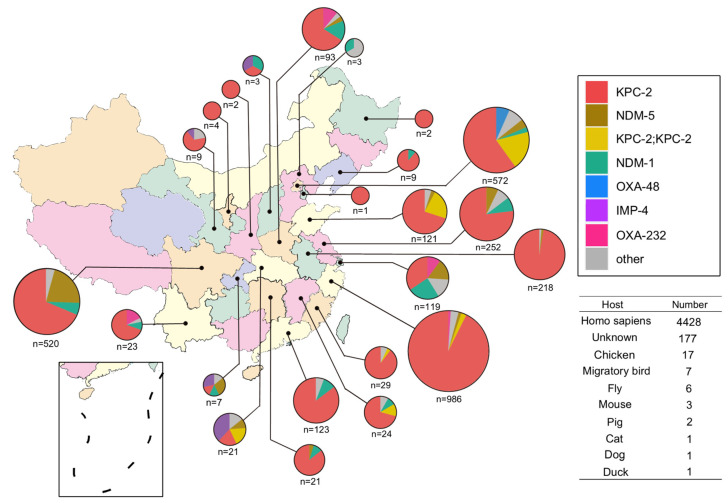
Carbapenemase type and geographical distribution of carbapenemase-carrying KPN.

As of April 20, 2022, the genomic data for a total of 37,322 strains of KPN have been downloaded. The data for *Klebsiella quasipneumoniae* and *Klebsiella variicola* were excluded from the analysis results of Kleborate Species, and the data for 37,264 KPN strains were collected. Through the collection of BioSample information, a total of 6107 KPN strains submitted by China were obtained by screening, of which 4643 were carbapenemase-carrying strains. The strain collection spanned from 2008 to 2022. The strain collection spanned from 2008 to 2022. A total of 3162 strains have provincial distribution information and are distributed in 23 provinces or municipalities directly under the central government. The three regions with the most genomic data were Zhejiang Province (986 strains), Beijing City (572 strains), and Sichuan Province (520 strains). A total of 4428 KPN strains were derived from Homo sapiens and 38 from mammals, poultry, and insects (Figure 1). We acknowledge the limitations of these genome sequencing data, and it is difficult to avoid selection bias from researchers in the selection of sequencing strains. Therefore, we chose to span a wider range of regions and times and use the phenotype of carbapenem to minimize data bias.

**Figure 2:**
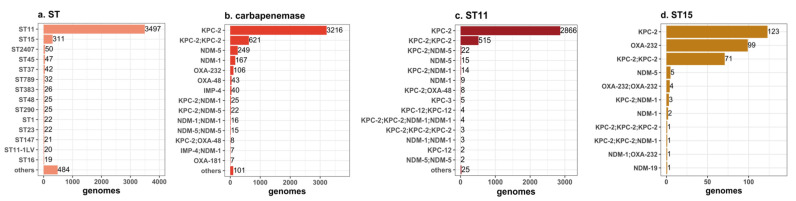
ST types (**a**) and carbapenemase types of KPN (**b**–**d**).

There are 152 ST types of carbapenemase-carrying KPN, of which the dominant types are ST11 (3497, 75.3%), ST15 (311, 6.7%), ST2407 (50, 1.1%), ST45 (47, 1.0%), and ST37 (42, 0.9%) (Figure 2a). Fifty-nine strains had polymorphism variations of a single housekeeping gene, five strains had gene polymorphism of two housekeeping genes, and one strain had gene polymorphism of three housekeeping genes.

### 2.1. Antimicrobial Resistance

Among these carbapenemase-carrying KPN, KPC-2 was the dominant enzyme type (3841/4643, 82.7%), including 3216 strains of KPC-2, 621 strains of KPC-2-KPC-2, and 4 strains of KPC-2-KPC-2-KPC-2. The NDM family was the second largest enzyme type, dominated by NDM-5 and NDM-1. NDM-5 (264/4643, 5.7%) included 15 strains of NDM-5-NDM-5. NDM-1 (183/4643, 3.9%) included 16 strains of NDM-1-NDM-1. The third largest enzyme type was the OXA family, dominated by OXA-232 (110/4643, 2.4%) and OXA-48 (43/4643, 0.9%). OXA-232 included 4 strains of OXA-232-OXA-232. The fourth largest enzyme type was IPM-4 (44/4643, 0.9%), which included 4 strains of IMP-4-IMP-4 (Figure 2b). Furthermore, there were a few combinations of serine enzymes and metalloenzymes, such as KPC-2-NDM-1 (25 strains), KPC-2-NDM-5 (22 strains), and KPC-2-OXA48 (8 strains), and metalloenzyme combinations, such as IMP-4-NDM-1 (7 strains). The main enzyme types of ST11 were KPC-2 (2866/3497, 82.0%) and KPC-2-KPC-2 (515/3497, 14.7%) (Figure 2c). ST15 was dominated by KPC-2 (123/311, 39.5%), OXA-232 (99/311, 31.8%), and KPC-2-KPC-2 (71/311, 22.8%) (Figure 2d).

KPC-2 had ST11 as the dominant clonotype (3384/3841, 88.1%), followed by ST15 (195/3841, 5.1%). OXA-232 had ST15 as the dominant clonotype (103/110, 93.6%), whereas OXA-48 had ST383 (25/43, 58.1%) and ST147 (8/43, 18.6%) as the dominant clonotypes. In contrast, NDM-5, NDM-1, and IMP-4 were spread among a wider range of clonotypes with no dominant clonotype. For example, 265 strains of NDM-5 were found in 60 ST types, with the highest proportion of ST2407 (49/265, 18.4%); 167 strains of NDM-1 were found in 58 ST types, with the highest proportion of ST37 (20 /167, 12.0%), and 44 strains of IPM-4 were found in 28 ST types, with the highest proportion of ST268 (6/44, 13.6%).

Observation results from different provinces and municipalities indicated that ST11 was the most common serotype in all regions, and the types of carbapenemase were slightly different in different regions. For example, KPC-2 was the absolute dominant type in Zhejiang Province (933/986, 94.6%); there were a total of 43 OXA-48 strains, of which 38 strains (88.4%) were detected in Beijing; there were 249 NDM-5 strains, of which 111 strains (44.6%) were detected in Sichuan Province. The three regions with more than 500 strains were Zhejiang Province (986 strains), with a KL64:KL47 ratio of 556:225; Beijing (572 strains), with a KL64:KL47 ratio of 105:337; and Sichuan Province (520 strains), with a KL64:KL47:KL25 ratio of 243:65:46.

Of the 1464 strains of carbapenemase-free KPN, 1195 strains (81.6%) had no OmpK35 OmpK36 mutation. A total of 81 types of mutants appeared, dominated by OmpK35-17% OmpK36GD (56 strains, 3.8%). Among the 4643 strains of carbapenemase-carrying KPN, there were 98 types of OmpK35 OmpK36 mutants, dominated by OmpK35-17% OmpK36GD (3376 strains, 72.7%). There were 720 strains (15.5%) that had no OmpK35 OmpK36 mutation.

There were 125 strains of KPN that carried genes related to polymyxin resistance, of which 94 strains carried *mgrB* mutations of a two-component system, 4 strains carried *pmrB* mutations of a two-component system, 10 strains carried the *mcr-1* gene, of which 1 strain was an imperfect hit (either <100% identity or <100% coverage), 11 strains carried *mcr-8*, of which 6 strains were imperfect hits, 2 strains carried two *mcr-8* imperfect hits, and 4 strains carried *mcr-9*. A total of 21 strains carried genes related to tigecycline resistance, of which 1 strain carried modification enzyme Tet(X4), and 20 strains carried RND-type pump gene clusters, including 2 strains of *tmexCD1-toprJ1 (mcr-8*, NDM-5), 16 strains of *tmexCD1-toprJ1*, and 2 strains carried two *tmexCD1-toprJ1* imperfect hits.

**Figure 3:**
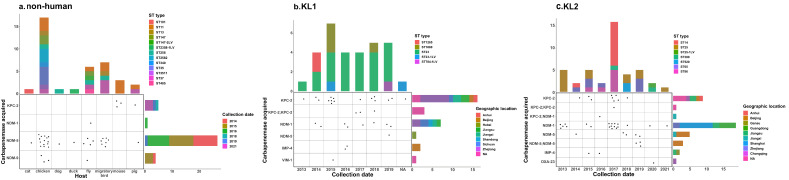
Distribution of carbapenemase-carrying non-human, KL1, and KL2 KPN. (**a**) Carbapenemase-carrying non-human KPN; (**b**) KL1 carbapenemase-carrying KPN; (**c**) KL2 carbapenemase-carrying KPN.

Of 4643 strains of carbapenemase-carrying KPN, 4428 strains (95.3%) were from humans, 177 strains (3.8%) were from unknown origin, and 38 strains (0.8%) were non-human (17 from chickens, 7 from migratory birds, 6 from flies, and 3 from mice). Of the 38 non-human-sourced strains, NDM metalloenzymes were the most common, with 29 strains of NDM-5 (76.3%), 4 strains of NDM-9 (10.5%), 4 strains of KPC-2 (10.5%), and 1 strain of NDM-1 (2.6%). (Figure 3a). Twenty-two of these strains were from chickens, dogs, and flies on Shandong chicken farms.

### 2.2. Virulence

#### 2.2.1. Aerobactin

The detection ratio of antimicrobial-sensitive and antimicrobial-resistant KL1 aerobactins nearly had no change, and they were all dominated by the *iuc-1* lineage. The proportion of antimicrobial-resistant KL2 *iuc-1* was obviously changed compared with that of sensitive KL2, decreasing to 26.1%, 50.0% of which had no *iuc-1*. In the antimicrobial-resistant ST11-CRKP, 48.4% of the strains contained *iuc-1*, and 41.4% of ST15-CRKP contained *iuc-1* (Table 1).

#### 2.2.2. Colibactin

Both antimicrobial-sensitive and antimicrobial-resistant KL1 colibactins were dominated by the *clb 2* lineage. Most KL2s, regardless of being antimicrobial-sensitive or antimicrobial-resistant, had no colibactin. The few strains with colibactin had clb 3. Both antimicrobial-sensitive and antimicrobial-resistant CKP were rarely detected with colibactin (Table 1).

#### 2.2.3. Salmochelin

The detection results of salmochelin were quite similar to those of aerobactin. Both antimicrobial-sensitive and antimicrobial-resistant KL1s were dominated by the iro 1 lineage, and the detection ratio of antimicrobial-resistant KL2 *iro 1* was obviously decreased, and most of them had no salmochelin. Salmochelin was not detected in most of the antimicrobial-sensitive and antimicrobial-resistant CKP (Table 1).

#### 2.2.4. Yersiniabactin

Both antimicrobial-sensitive and antimicrobial-resistant KL1 yersiniabactins were dominated by the *ybt1* ICEKp10 lineage. Yersiniabactin was not detected in many strains of antimicrobial-sensitive and antimicrobial-resistant KL2, and those with yersiniabactin also showed diverse lineages. Sensitive CKP mostly lacked yersiniabactin. ST11-CRKP was dominated by *ybt9* ICEKp3, and ST15-CRKP had diverse yersiniabactin lineages (Table 1).

#### 2.2.5. RmpADC

The detection results of RmpADC were similar to those of aerobactin and salmochelin. Both antimicrobial-sensitive and antimicrobial-resistant KL1s were dominated by the *rmp1* KpVP-1 lineage. In antimicrobial-resistant KL2, the detection ratio of *rmp1* KpVP-1 obviously decreased. Antimicrobial-sensitive and antimicrobial-resistant CKP were mostly free of *rmpADC*, especially ST15-CRKP, which had no *rmpADC*, while ST11-CRKP had a 26.7% detection ratio of *rmp1* KpVP-1. Sensitive CKP had a 6.8% detection rate of *rmp1* KpVP-1 (Table 1).

### 2.3. Serotype

Of the 31 strains of KL1, the dominant serotype was ST23 (22/31, 70.9%) and carried carbapenemase KPC-2 (19/31, 61.3%) predominantly. The first detected strain of KL1-CRHvKP was from the ST23-type strains of CTX-M-55 and KPC-2, carried by a patient from Zhejiang (see Figure 3b). The 42 strains of KL2 had diverse ST types, with ST25 (12/42, 28.5%), ST14 (11/42, 26.1%), ST65 (7/42, 16.6%), and ST86 (7/42, 16.6%) being the most prevalent. The carbapenemases carried were mainly NDM-1 (20/42, 47.6%) and KPC-2 (10/42, 23.8%), and one strain carried both KPC-2 and NDM-1. The earliest KL2-CRHvKP strain appeared in the ST25 strain carrying NDM-1 in Guangdong patients in 2013 (Figure 3c).

**Figure 4:**
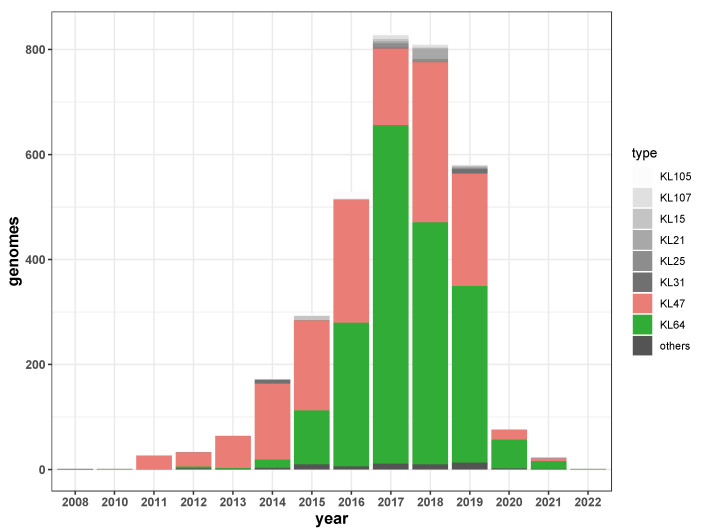
Serotype distribution of ST11 carbapenemase-carrying KPN.

The most common capsular serotypes of ST11 were KL64 (1929/3497, 55.2%) and KL47 (1388/3497, 39.7%). Interestingly, a reversal occurred in the proportions of these two dominant clones. ST11-KL47 appeared in China in 2010, and ST11-KL64 appeared in 2012. KL47 was dominant from 2010 to 2014. In 2015, the dominance of KL47 decreased (171/296, 57.8%), and the proportion of KL64 rose rapidly (103/296, 34.8%). In 2016, the proportions of KL64 (274/529, 51.8%) and KL47 (234/529, 44.2%) reversed. In subsequent years, although KL64 remained dominant, KL47 did not disappear but remained the second most prevalent clone (Figure 4). KL19 (146/311, 46.9%), KL112 (111/311, 35.7%), and KL24 (34/311, 10.9%) were the dominant capsular serotypes of ST15.

## 3. Discussion

Multidrug-resistant CKP clones are highly diverse, with whole-genome diversity driven by frequent gains and losses of plasmids and phages. Capsular polysaccharide diversity results from chromosomal recombination. The highly virulent clones are seldom subject to chromosomal recombination and therefore have low plasmid and whole-genome diversity [22]. Furthermore, capsular serotypes seldom deviate from KL1, KL2, and KL5 [1]. This may be due to the overproduction of capsular polysaccharides in many HvKP strains interfering with DNA uptake [23]. In this study, the carbapenemase-carrying KPN had 90 capsular serotypes. With the exception of ST11 and ST15, a total of 835 strains were dispersed among 150 ST types. There were also 26 polymorphic variants consisting of one, two, and three housekeeping genes. This indicates that carbapenemases were widely transferred between KPN subtypes. In China, carbapenemase resistance genes have been transferred to community-based hypervirulent KL1 and KL2 HvKP since 2013. KL1 is dominated by KPC-2, and KL2 is dominated by NDM-1, followed by KPC-2. Since 2014, the propagation of carbapenemase into chickens, migratory birds, flies, mice, and pigs has been detected, with metalloenzyme NDM-5 being the most common.

The ST11 capsular serotype had a reversal of the prevalence of KL47 and KL64 in 2016, with KL64 replacing KL47 as the dominant serotype. CRKP KL47 and KL64 prevalence were also reversed in 2016 in bloodstream infection in a tertiary hospital in China, with KL64 gradually replacing KL47 [24]. In this study, the data of carbapenemase-carrying KPN from many regions of China showed that although the proportion of KL47 decreased, it is still the second largest capsular serotype and has not been eliminated.

Colibactin is a genotoxin expressed by the pks genomic island (*clbA-clbS*). It is associated with DNA double-strand breaks, chromosomal abnormalities, and cell cycle arrest. The survey data of a tertiary university hospital in Egypt showed that KL1 was greatly correlated with the co-carrying of colibactin [25]. A total of 66% of colibactin-positive KPN strains in Taiwan are of KL1 type [26]. The data in this study showed that colibactin can distinguish hypervirulent KL1 from classic KPN, making colibactin a suitable indicator for discriminating between KL1-type HvKP and CKP. In contrast, most KL2 does not carry colibactin. Similar to previous findings [14], KL2 is genetically more diverse than KL1. In this study, of the 6107 strains of KPN from China, a total of 336 strains were KL1, and 187 strains were KL2. KL1 had 287 strains (85.4%) of KL1 were ST23, whereas KL2 had 14 ST types, with the most prevalent ST65 accounting for 59 strains (31.6%). Resistant carbapenemase-carrying KL2 showed a large decrease in aerobactin, salmochelin, and *rmpADC* compared with sensitive KL2, which was associated with the different ST-type composition ratios of resistant and sensitive KL2. For antimicrobial-resistant KL2 KPN, ST14 and ST25 were more prevalent (23 strains, 54.8%), and ST65 and ST86 were less prevalent (14 strains, 33.3%). In contrast, for sensitive KL2 KPN, ST65 and ST86 were more prevalent (77 strains, 59.7%), and ST14 and ST25 were less prevalent (22 strains,17.1%). ST65 and ST86 carried more virulence genes than ST14 and ST25. The virulence genes of KL1-CRHvKP and ST65 and ST86 KL2-CRHvKP were not lost after carbapenemase was obtained.

Iron acquisition is a key virulence factor for HvKP, the siderophore production of which increased by six- to ten-fold compared with CKP [27]. Among the siderophores of aerobactin, yersiniabactin, and salmochelin, aerobactin is considered the decisive genetic feature and potential antivirulence target of HvKP [28]. However, the data of this study showed that 48.4% of ST11-CRKP and 41.4% of ST15-CRKP also carried the iuc-1 lineage, which was consistent with the KL1 lineage. ICEKp is the most common mobile genetic element in KPN and is usually integrated at an aspartate (Asn) tRNA site. The Ybt locus is often mobilized by ICEKp, and ybt and ICEKp are diverse. Each ICEKp carries a different cargo gene cluster at the right end, and ICEKp10 often carries the clb locus and is associated with ybt lineages 1, 12, and 17 [17]. In this study, the common yersiniabactin in KL1 was ybt1 ICEKp10, the most common yersiniabactin in ST11-CRKP was ybt9 ICEKp3, and KL2 and ST15-CRKP were represented by diverse ybt and ICEKp.

OmpK35 and OmpK36 are the major non-specific porins of KPN, through which nutrients and hydrophilic β-lactam antibiotics can diffuse across the membrane [29,30]. Their most common mutations are premature emergence of stop codons and truncated frameshifts, and strains lacking these two porins have enhanced antimicrobial resistance [31]. The existence of porin deletion, along with carbapenem, is often reflected as high levels of resistance. There are many heterogeneities in the truncation and mutation of OmpK35 and OmpK36. In this study, 4643 carbapenem-carrying KPN strains had 98 types of mutants, 6107 strains of KPN from China had 146 types of mutants, and 37,264 strains of KPN had 651 types of mutants. Although there are many types of mutations, there are only a few prevalent mutants. OmpK35-17% OmpK36GD was the most common (3893/37,264, 10.4%), of which 89.4% (3482/3893) were type ST11. The second most prevalent mutant was OmpK35-25% (3518/37,264, 9.4%), of which 86.0% are ST258. The truncation and mutation of OmpK35 and OmpK36 are spontaneous mutations, and their coexistence with carbapenemase is largely related to the spread of regional dominant clones.

In this study, 79.2% of OXA-48 was detected in Beijing and 44.6% of NDM-5 in Sichuan Province. Unlike other regions, KL47 was the predominant capsular serotype in Beijing. Although the data in this study covered 23 provinces or municipalities across China, the amount of data in each region varied greatly, making it difficult to analyze differences in molecular epidemiology between regions.

## 4. Materials and Methods

### 4.1. Klebsiella pneumoniae Genome-Wide Data

The *Klebsiella pneumoniae* genome-wide data as of 20 April 2022, were downloaded from the NCBI Assembly database.

### 4.2. Kleborate Analysis

All *Klebsiella pneumoniae* genomes were analyzed with Kleborate (https://github.com/katholt/Kleborate, accessed on from 25 August 2021 to 20 April 2022, multiple times). Kleborate analysis modules include assembly quality, species, multi-locus sequence typing (MLST), acquired virulence determinants, serotype prediction, and antimicrobial resistance determinants [32].

### 4.3. BioSample Information Collection

All BioSample information identified as KPN by Species was collected, including collection date, geographic location, host, and isolation source.

### 4.4. Comparative Analysis of Virulence Genes

The virulence genes (aerobactin, colibactin, salmochelin, yersiniabactin, and *rmpADC*) of five groups of KPN were compared, which included KL1 (270 strains) and KL2 (129 strains) HvKP; KL1 (31 strains) and KL2 (42 strains) CRHvKP; antimicrobial-sensitive (ESBL-; Carb-) CKP (366 strains); and the two types of CRKP with the highest prevalence, ST11-CRKP (3497 strains), and ST15-CRKP (311 strains).

## 5. Conclusions

Through this large sample molecular epidemiological analysis of carbapenemase-carrying KPN in China, we found that carbapenemases have been widely transferred in CKP subtypes and are infiltrating into KL1, KL2 HvKP, and non-human hosts. The KL1 and KL2 HvKP acquire carbapenemases, which are truly carbapenem-resistant and hypervirulent strains, posing a serious threat to the survival rate of infected hosts. It is imperative to limit transmission among human hosts, non-human hosts, and environments. In addition to antimicrobial resistance, monitoring of high virulence should also be given attention. We must take measures to strengthen the colonization screening of antibiotic-resistant and/or highly virulent strains in hospitalized patients, conduct regular screening of farm animals and the environment, and rationally use antibiotics when infection is indicated. The definition and screening indicators of high-virulence KPN have been controversial in academic circles. In this large sample analysis, we found that colibactin can be used as an indicator to distinguish KL1 HvKP from CKP.

## Figures and Tables

**Table 1:** Virulence genes of HvKP and CKP with antimicrobial sensitivity and resistance.

Species	Aerobactin (*iuc* Lineage)			Colibactin (*clb* Lineage)			Salmochelin (*iro* Lineage)			Yersiniabactin *(ybt* Lineage)			RmpADC *(rmp* Lineage)		
	Type	No.	Prevalence	Type	No.	Prevalence	Type	No.	Prevalence	Type	No.	Prevalence	Type	No.	Prevalence
KL1 (270)	*iuc-1*	242	89.6%	*clb 2*	226	83.7%	*iro 1*	234	86.6%	*ybt1*; ICEKp10	239	88.5%	*rmp1*; KpVP-1	225	83.3%
ESBL− ^a^;Carb− ^b^	-	20	7.4%	-	28	10.3%	-	17	6.2%	*ybt2*; ICEKp1	17	6.2%	-	24	8.8%
													*rmp3*; ICEKp1	14	5.1%
KL1 (31)	*iuc-1*	28	90.3%	*clb 2*	30	96.7%	*iro 1*	27	87.1%	*ybt1*; ICEKp10	30	96.7%	*rmp1*; KpVP-1	25	80.6%
Carb+ ^c^	-	3	9.7%	-	1	3.2%	-	4	12.9%				-	4	12.9%
KL2 (129)	*iuc-1*	98	75.9%	-	84	65.1%	*iro 1*	101	78.2%	-	57	44.1%	*rmp1*; KpVP-1	95	73.6%
ESBL−; Carb−	-	17	13.1%	*clb 3*	35	27.1%	-	14	10.8%	*ybt17*; ICEKp10	36	27.9%	-	16	12.4%
	*iuc-2*	9	6.9%	*clb 1*	8	6.2%	*iro 2*	9	6.9%	*ybt9*; ICEKp3	9	6.9%	*rmp2*; KpVP-2	9	6.9%
KL2 (42)	-	21	50.0%	-	36	85.7%	-	26	61.9%	-	16	38.0%	-	26	61.9%
Carb+	*iuc-1*	11	26.1%	*clb 3*	5	11.9%	*iro 1*	13	30.9%	*ybt16*; ICEKp12	10	23.8%	*rmp1*; KpVP-1	13	30.9%
	*iuc-3*	7	16.6%							*ybt17*; ICEKp10	5	11.9%			
CKP (366)	-	302	82.5%	-	350	95.6%	-	318	86.8%	-	237	64.7%	-	326	89.0%
ESBL−; Carb−	*iuc-1*	31	8.4%				*iro 1*	32	8.7%	*ybt9*; ICEKp3	33	9.0%	*rmp1*; KpVP-1	25	6.8%
	*iuc-3*	13	3.5%							*ybt10*; ICEKp4	20	5.4%			
ST11-CRKP (3497)	-	1764	50.4%	-	3496	99.9%	-	3302	94.4%	*ybt9*; ICEKp3	3400	97.2%	-	2460	70.3%
Carb+	*iuc-1*	1694	48.4%							-	38	1.0%	*rmp1*; KpVP-1	936	26.7%
ST15-CRKP (311)	-	174	55.9%	-	311	100.0%	-	310	99.6%	*ybt14*; ICEKp5	144	46.3%	-	311	100.0%
Carb+	*iuc-1*	129	41.4%							*ybt16*; ICEKp12	102	32.7%			
										*ybt14*; ICEKp12	30	9.6%			

^a^ Negative of ESBL genes; ^b^ negative of carbapenemase; ^c^ positive of carbapenemase.

## Data Availability

All genome data in this article are available on the NCBI website at https://www.ncbi.nlm. nih.gov/assembly/ (accessed on from 25 August 2021 to 20 April 2022, multiple times).

## References

[1] Wyres K.L., Lam M., Holt K.E. (2020). Population genomics of Klebsiella pneumoniae. Nat. Rev. Microbiol..

[2] Podschun R., Ullmann U. (1998). Klebsiella spp. as nosocomial pathogens: Epidemiology, taxonomy, typing methods, and pathogenicity factors. Clin. Microbiol. Rev..

[3] Gorrie C.L., Mirčeta M., Wick R.R., Edwards D.J., Thomson N.R., Strugnell R.A., Pratt N.F., Garlick J.S., Watson K.M., Pilcher D.V. (2017). Gastrointestinal carriage is a major reservoir of Klebsiella pneumoniae infection in intensive care patients. Clin. Infect. Dis..

[4] Jones R.N. (2010). Microbial etiologies of hospital-acquired bacterial pneumonia and ventilator-associated bacterial pneumonia. Clin. Infect. Dis..

[5] Tang X.-J., Sun B., Ding X., Li H., Feng X. (2020). Changing trends in the bacteriological profiles and antibiotic susceptibility in neonatal sepsis at a tertiary children’s hospital of China. Transl. Pediatrics.

[6] Shon A.S., Bajwa RP S., Russo T.A. (2013). Hypervirulent (hypermucoviscous) Klebsiella pneumoniae: A new and dangerous breed. Virulence.

[7] Wyres K.L., Holt K.E. (2018). Klebsiella pneumoniae as a key trafficker of drug resistance genes from environmental to clinically important bacteria. Curr. Opin. Microbiol..

[8] Guh A.Y., Bulens S.N., Mu Y., Jacob J.T., Reno J., Scott J., Wilson L.E., Vaeth E., Lynfield R., Shaw K.M. (2015). Epidemiology of carbapenem-resistant Enterobacteriaceae in 7 US communities, 2012–2013. JAMA.

[9] Grundmann H., Glasner C., Albiger B., Aanensen D.M., Tomlinson C.T., Andrasević A.T., Cantón R., Carmeli Y., Friedrich A.W., Giske C.G. (2017). Occurrence of carbapenemase-producing Klebsiella pneumoniae and Escherichia coli in the European survey of carbapenemase-producing Enterobacteriaceae (EuSCAPE): A prospective, multinational study. Lancet Infect. Dis..

[10] Brisse S., Fevre C., Passet V., Issenhuth-Jeanjean S., Tournebize R., Diancourt L., Grimont P. (2009). Virulent clones of Klebsiella pneumoniae: Identification and evolutionary scenario based on genomic and phenotypic characterization. PLoS ONE.

[11] Luo Y., Wang Y., Ye L., Yang J. (2014). Molecular epidemiology and virulence factors of pyogenic liver abscess causing Klebsiella pneumoniae in China. Clin. Microbiol. Infect..

[12] Liao C.-H., Huang Y.T., Chang C.Y., Hsu H.S., Hsueh P.-R. (2014). Capsular serotypes and multilocus sequence types of bacteremic Klebsiella pneumoniae isolates associated with different types of infections. Eur. J. Clin. Microbiol. Infect. Dis..

[13] Bialek-Davenet S., Criscuolo A., Ailloud F., Passet V., Jones L., Delannoy-Vieillard A.-S., Garin B., Le Hello S., Arlet G., Nicolas-Chanoine M.-H. (2014). Genomic definition of hypervirulent and multidrug-resistant Klebsiella pneumoniae clonal groups. Emerg. Infect. Dis..

[14] Struve C., Roe C.C., Stegger M., Stahlhut S.G., Hansen D.S., Engelthaler D.M., Andersen P.S., Driebe E.M., Keim P., Krogfelt K.A. (2015). Mapping the evolution of hypervirulent Klebsiella pneumoniae. MBio.

[15] Holt K.E., Wertheim H., Zadoks R.N., Baker S., Whitehouse C.A., Dance D., Jenney A., Connor T.R., Hsu L.Y., Severin J. (2015). Genomic analysis of diversity, population structure, virulence, and antimicrobial resistance in Klebsiella pneumoniae, an urgent threat to public health. Proc. Natl. Acad. Sci. USA.

[16] Lam M.M.C., Wyres K.L., Judd L.M., Wick R.R., Jenney A., Brisse S., Holt K.E. (2018). Tracking key virulence loci encoding aerobactin and salmochelin siderophore synthesis in Klebsiella pneumoniae. Genome Med..

[17] Lam M.M.C., Wyres K.L., Judd L.M., Wick R.R., Jenney A., Brisse S., Holt K.E. (2018). Genetic diversity, mobilisation and spread of the yersiniabactin-encoding mobile element ICEKp in Klebsiella pneumoniae populations. Microb. Genom..

[18] Walker K.A., Miner T.A., Palacios M., Trzilova D., Frederick D.R., Broberg C., Sepúlveda V.E., Quinn J., Miller V.L. (2019). A Klebsiella pneumoniae regulatory mutant has reduced capsule expression but retains hypermucoviscosity. MBio.

[19] Walker K.A., Treat L.P., Sepúlveda V.E., Miller V.L. (2020). The small protein RmpD drives hypermucoviscosity in Klebsiella pneumoniae. MBio.

[20] Zhang R., Lin D., Chan E.W.-C., Gu D., Chen G.-X., Chen S. (2016). Emergence of carbapenem-resistant serotype K1 hypervirulent Klebsiella pneumoniae strains in China. Antimicrob. Agents Chemother..

[21] Gu D., Dong N., Zheng Z., Lin D., Huang M., Wang L., Chan E.W.-C., Shu L., Yu J., Zhang R. (2018). A fatal outbreak of ST11 carbapenem-resistant hypervirulent Klebsiella pneumoniae in a Chinese hospital: A molecular epidemiological study. Lancet Infect. Dis..

[22] Gonzalez-Ferrer S., Peñaloza H.F., Budnick J.A., Bain W.G., Nordstrom H.R., Lee J.S., Van Tyne D. (2021). Finding order in the chaos: Outstanding questions in Klebsiella pneumoniae pathogenesis. Infect. Immun..

[23] Wyres K.L., Wick R.R., Judd L.M., Froumine R., Tokolyi A., Gorrie C.L., Lam M.M.C., Duchêne S., Jenney A., Holt K.E. (2019). Distinct evolutionary dynamics of horizontal gene transfer in drug resistant and virulent clones of Klebsiella pneumoniae. PLoS Genet..

[24] Zhou K., Xiao T., David S., Wang Q., Zhou Y., Guo L., Aanensen D., Holt K.E., Thomson N.R., Grundmann H. (2020). Novel subclone of carbapenem-resistant Klebsiella pneumoniae sequence type 11 with enhanced virulence and transmissibility, China. Emerg. Infect. Dis..

[25] El-Ashry A.H., Hendawy S.R., Mahmoud N.M. (2022). Prevalence of pks genotoxin among hospital-acquired Klebsiella pneumoniae. AIMS Microbiol..

[26] Lai Y.-C., Lin A.-C., Chiang M.-K., Dai Y.-H., Hsu C.-C., Lu M.-C., Liau C.-Y., Chen Y.-T. (2014). Genotoxic Klebsiella pneumoniae in Taiwan. PLoS ONE.

[27] Russo T.A., Olson R., MacDonald U., Metzger D., Maltese L.M., Drake E.J., Gulick A. (2014). Aerobactin mediates virulence and accounts for increased siderophore production under iron-limiting conditions by hypervirulent (hypermucoviscous) Klebsiella pneumoniae. Infect. Immun..

[28] Russo T.A., Olson R., MacDonald U., Beanan J., Davidson B.A. (2015). Aerobactin, but not yersiniabactin, salmochelin, or enterobactin, enables the growth/survival of hypervirulent (hypermucoviscous) Klebsiella pneumoniae Ex Vivo and In Vivo. Infect. Immun..

[29] Sugawara E., Kojima S., Nikaido H. (2016). Klebsiella pneumoniae major porins OmpK35 and OmpK36 allow more efficient diffusion of β-lactams than their Escherichia coli homologs OmpF and OmpC. J. Bacteriol..

[30] Wong J.L.C., Romano M., Kerry L.E., Kwong H.-S., Low W.-W., Brett S.J., Clements A., Beis K., Frankel G. (2019). OmpK36-mediated carbapenem resistance attenuates ST258 Klebsiella pneumoniae in vivo. Nat. Commun..

[31] Tsai Y.-K., Fung C.-P., Lin J.-C., Chen J.-H., Chang F.-Y., Chen T.-L., Siu L.K. (2011). Klebsiella pneumoniae outer membrane porins OmpK35 and OmpK36 play roles in both antimicrobial resistance and virulence. Antimicrob. Agents Chemother..

[32] Lam M.M.C., Wick R.R., Watts S.C., Cerdeira L.T., Wyres K.L., Holt K.E. (2021). A genomic surveillance framework and genotyping tool for Klebsiella pneumoniae and its related species complex. Nat. Commun..

